# Functional characterization of FvCAMTA1in salt stress response of *Fraxinus velutina*

**DOI:** 10.3389/fpls.2025.1669043

**Published:** 2025-10-29

**Authors:** Liping Yan, Dali Geng, Yinhua Wang, Chao Sun, Tianjiao Li, Baizhu Wang, Junxiu Yao, Fei Ren, Lianjia Yu

**Affiliations:** ^1^ Shandong Provincial Academy of Forestry, Jinan, China; ^2^ School of Forestry Engineering, Shandong Agriculture and Engineering University, Zibo, China

**Keywords:** *Fraxinus velutina*, FvCAMTA1, salt stress, calcium signaling, transcription factor

## Abstract

The calmodulin-binding transcription activator (CAMTA) family plays crucial roles in calcium-mediated abiotic stress responses in plants. This study isolated and functionally characterized *FvCAMTA1*, a CAMTA gene from the salt-tolerant woody species *Fraxinus velutina*. Promoter analysis identified salt-responsive cis-elements, with a 157-bp core region sufficient for basal promoter activity and upstream sequences enhancing transcriptional activation under salt stress. *FvCAMTA1* was predominantly expressed in leaves and rapidly induced by NaCl treatment. The heterologous overexpression of *FvCAMTA1* in *Arabidopsis* significantly enhanced salt tolerance, resulting in higher germination rates, improved root elongation, and increased fresh weight, whereas the *camta5* mutant exhibited heightened sensitivity. Yeast two-hybrid screening identified 46 proteins interacting with *FvCAMTA1*, including *FvWRKY7* and *FvPP2C60*, interactions subsequently confirmed by bimolecular fluorescence complementation and luciferase complementation assays. Our findings demonstrate that *FvCAMTA1* acts as a positive regulator in the salt stress adaptation of woody plants through calcium signaling and transcriptional networks, providing a valuable candidate gene for molecular breeding of stress-resistant trees.

## Introduction

Calmodulin (CaM), an essential Ca^2+^ signal sensor in plant cells, is a highly conserved calcium-binding protein that widely participates in plant growth, development, and environmental adaptation processes (Furio et al., 2020). Molecular interaction studies have identified over 90 calmodulin-binding transcription factors, including members of the CAMTA, MYB, bZIP, NAC, CBP60, WRKY, and MADS-box families (Park et al., 2005; Finkler et al., 2007; Popescu et al., 2007; Reddy et al., 2011; Singh et al., 2013; Zeng et al., 2015). Among these, the calmodulin-binding transcription activator (CAMTA) family has been confirmed as core regulatory components in plant Ca^2+^/CaM signaling pathways due to their unique calcium responsiveness (Yang et al., 2015; Kakar et al., 2018). Research on CAMTA transcription factors reveals their multifaceted roles in plant growth and abiotic stress responses through calcium signaling, including plant hormone response regulation, drought stress response, cold stress mediation, and immune response regulation. *NtER1*, a CAMTA family member, is induced by ethylene and participates in regulating plant senescence (Yang and Klee, 2000); Arabidopsis *CAMTA1* mediates auxin signaling pathways to respond to heat stress (Galon et al., 2010a, 2010b).

The *camta1* mutant exhibits reduced photosynthetic efficiency, water use efficiency, and relative water content under drought conditions, with significant alterations in gene expression related to osmoregulation, apoptosis, photosynthesis, and DNA methylation, suggesting *AtCAMTA1*’s involvement in drought recovery mechanisms (Pandey et al., 2013). The *Atcamta1/camta3* double mutant shows significantly reduced freezing tolerance, indicating synergistic regulation of cold responses by *AtCAMTA1* and *AtCAMTA3* (Doherty et al., 2009) Calcium-bound AtCAMTA3/SR1 activates plant immune responses through protein binding (Feys et al., 2001; Zhang et al., 2012). *AtCAMTA5* and *AtCAMTA6* participate in responses to cold (Kidokoro et al., 2017), water deprivation (Méndez-Gómez et al., 2024), and salt (Shkolnik et al., 2019; Hau et al., 2024). Despite evidence showing salt stress-induced Ca^2+^ signaling in *Arabidopsis* (Bouche et al., 2005; Kim et al., 2009; Mahajan et al., 2009), molecular mechanisms decoding calcium signals and downstream pathways in woody plants remain largely unknown.

*Fraxinus velutina*, a deciduous tree native to southwestern North America, exhibits rapid growth and exceptional salinity tolerance. Owing to these traits, it has been widely introduced for cultivation in saline soils of China’s Yellow River Delta (Mao et al., 2017). Nevertheless, the molecular mechanisms underpinning the high salt tolerance in *F. velutina* remain largely elusive. Our previous transcriptomic comparison between salt-tolerant *F. velutina* and salt-sensitive *F. chinensis* identified 316 salt-induced genes, including *FvCAMTA1*. This gene exhibits significant induction kinetics under NaCl treatment and structural conservation with herbaceous homologs. Here, we present comprehensive analyses of *FvCAMTA1* promoter architecture, expression dynamics, and functional validation through transgenic approaches. By integrating promoter truncation assays, and Arabidopsis transformation, this work provides mechanistic insights into CAMTA-regulated woody plant salt adaptation.

## Materials and methods

### Plant materials and treatments

Three-month old *Fraxinus velutina* tissue culture seedlings of variety ‘Lu Xiaowu 6’ and ‘Qing Bi’ from the Shandong Provincial Key Laboratory of Tree Breeding were used. Salt treatments involved hydroponic culture with 100-/300-mM NaCl solutions for 0, 1, 4, and 12 h. Then, fresh leaves were flash-frozen in liquid nitrogen for RNA extraction. *Arabidopsis* Col-0, *camta5* mutants, and transgenic lines were maintained under controlled conditions (16 h light/8 h dark, 22°C).

### Gene cloning and vector construction

Specific primers containing NdeI/KpnI sites amplified the coding sequence from leaf cDNA. The product was cloned into pMD18-T (TaKaRa) and verified by Sanger sequencing. For promoter analysis, three fragments (−1,084 to +1, −307 to +1, −157 to +1) were amplified using genome walking techniques and inserted into pPZP211-GUS vectors. Primers used for gene cloning are shown in **Supplementary Table 1**.

### Bioinformatic analysis

NCBI CD-Search, InterPro, and DNAMAN 9.0 identified conserved domains. NetWheels predicted α-helix patterns in CaMBD regions. Phylogenetic trees were constructed using MEGA5.1 (Neighbor-Joining method). ProtScale and SOPMA analyzed hydrophobicity and secondary structure.

### Tobacco transient expression and Arabidopsis transformation

Agrobacterium-mediated transient assays used 5-week-old *N. benthamiana* leaves. Infiltration buffer contained 10 mM MgCl_2_, 10 mM MES (pH 5.6), and 100 μM acetosyringone. GUS staining (75 mM Na_3_PO_4_ pH 7.0, 0.05 mM K_3_[Fe(CN)_6_], 50 μg/mL X-Gluc) followed 48 h post-infiltration. Stable Arabidopsis transformation employed the floral dip method. T3 homozygous lines were selected on 50 mg/L kanamycin.

### Salt stress treatment

*Arabidopsis* Col-0, *camta5* mutants, and transgenic line were used for salt stress treatment. Germination assays scored seeds on 1/2MS medium with 0/200/300 mM NaCl after 7 days. Root elongation measurements were done at 21-day treatments. Hydroponic experiments quantified fresh weight after 30-day 200 mM NaCl exposure. Three biological replicates were conducted for all assays.

### RNA extraction, qRT-PCR, yeast two-hybrid, BiFC, and LCA

Total RNA was extracted with the RNAprep Pure Plant Kit (#DP441, polysaccharides and polyphenolics-rich, Tiangen Biotech, Beijing, China). 2 µg of total RNA was used to synthesize the first-strand cDNA with the PrimeScript™ 1st Strand cDNA Synthesis Kit (Takara, Shiga, Japan). The cDNA reaction mixture was diluted five times, and 5 µl was used in the 20-µl PCR reaction. The PCR reactions included a pre-incubation step at 95°C for 2 min followed by 45 cycles of denaturation at 95°C for 15 s, annealing at 54°C for 30 s, and extension at 72°C for 30 s. All reactions were performed in the QuantStudio™ 5 Food Safety Real-Time PCR System using TB Green Fast qPCR Mix (Takara) and ROX reference dye. Each experiment had nine replicates (three technical replicates for each biological replicate). The relative expression level was calculated using the 2^^−ΔΔCt^ method.

Yeast two-hybrid screening and analysis were performed as methods mentioned by Chen and Wei (2022). A cDNA library was created by seeding of *Fraxinus velutina* with 150 mmol/L NaCl treated for 12 h. Bimolecular fluorescence complementation was performed as methods mentioned by Goto-Yamada et al. (2018). Luciferase complementation assay (LCA) was performed by methods mentioned by Zhou et al. (2018).

All primers used are listed in **Supplementary Table 1**.

### Microscopy and statistical analysis

Confocal imaging (Leica TCS SP5) visualized signals in transformed protoplasts. ImageJ quantified GUS staining intensity. Data were analyzed using SPSS 18.0 with ANOVA and Duncan’s multiple range tests (p<0.05).

## Results

### Molecular characterization of *FvCAMTA1*

**Figure 1:**
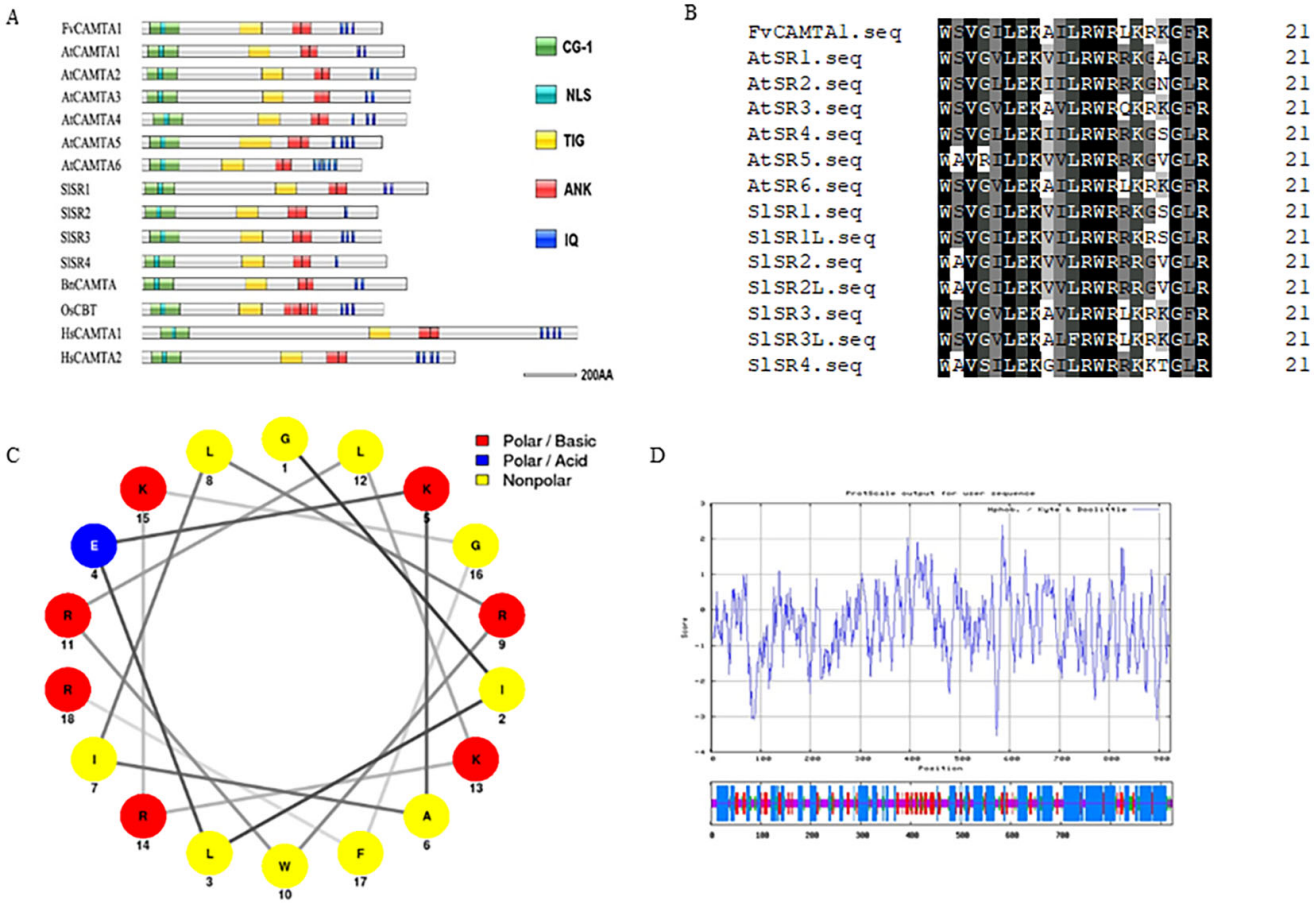
The domains and secondary structures of FVCAMTA1. **(A)** Multiple comparisons of FVCAMTA1 with CaMBD sequences from other species; **(B)** The a-helix diagram of the CaMBD sequence of FVCAMTA1. **(C)** The predicted results of the domains of CAMTAS; **(D)** The predicted results of the secondary structure of the FVCAMTA1 protein.

The 2,772-bp *FvCAMTA1* ORF encodes 923 amino acids with a 104.33-kDa molecular weight and pI 7.37. Conserved domain analysis identified the N-terminal CG-1 DNA-binding domain (residues 30-130) with a nuclear localization signal; the TIG interaction domain (residues 140-250); ANK repeats (residues 260-380); and C-terminal IQ motifs (Ile763-Arg815): IQ1 (763IQHAFRKYETK773), IQ2 (782IQYRFRTWKMR792), and IQ3 (805IQAAVRGFQVR815) (**Figures 1A, B**). NetWheels modeling revealed an amphipathic α-helix structure in the CaMBD region (residues 826-843), with hydrophobic residues clustered at 0°-180° and hydrophilic residues (Arg838, Lys839, Arg841) at 180°-360°, consistent with classical CaMBD patterns (**Figure 1C**). Secondary structure prediction showed 45.50% α-helix, 15.17% β-sheet, 9.10% β-turn, and 30.23% random coils (**Figure 1D**).

**Figure 2:**
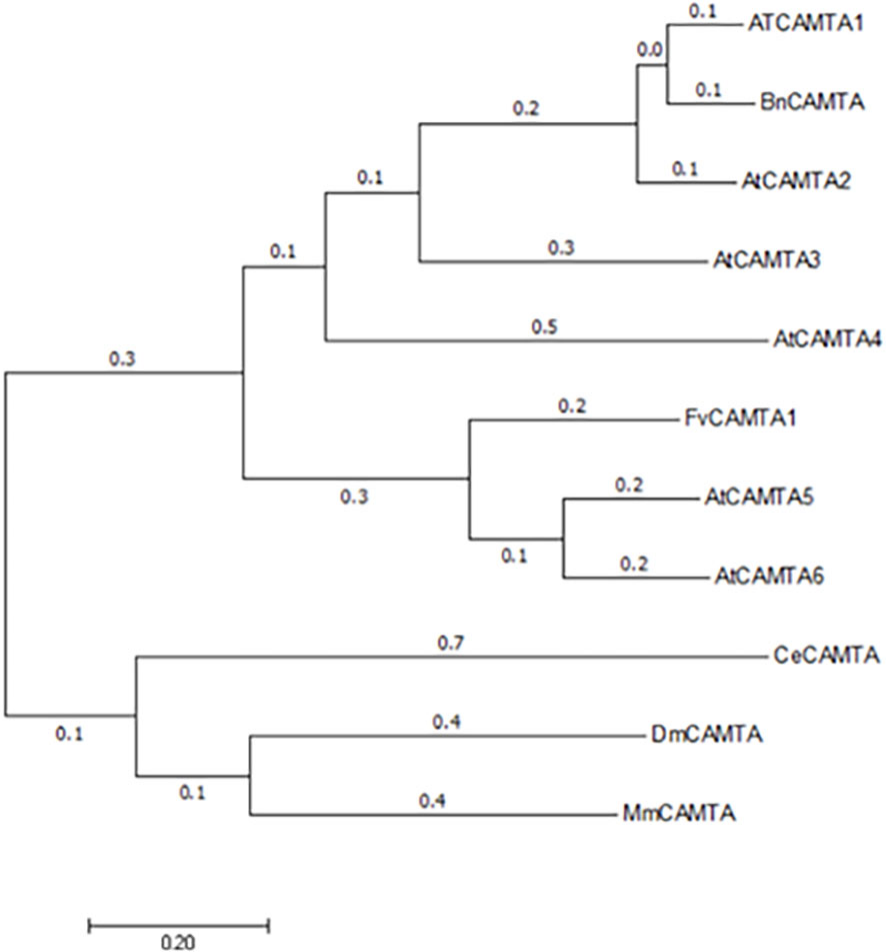
Construction of the phylogenetic tree of *FVCAMTAI*. The phylogenetic tree was constructed using the Neighbor-Joining method; the number of Bootstrap replications was 1000.

Neighbor-joining tree (Saitou and Nei 1987) of CAMTAs from multiple species grouped *FvCAMTA1* with *Arabidopsis AtCAMTA5* (82% identity) and tomato *SlCAMTA2* (**Figure 2**). These results indicating *FvCAMTA1* is a member of CATMA family.

### Promoter of *FvCAMTA1* contains salt-response elements

**Figure 3:**
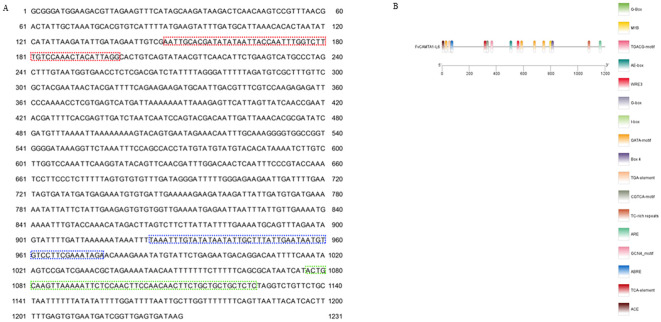
The *FVCAMTA1* promoter analysis. **(A)** The promoter sequence cloned in this study. The dotted box indicates three potential promoter sites predicted by the NNPP online database. **(B)** Predicted motifs by PlantCARE.

Through genome walking techniques, we obtained a 1,231-bp promoter sequence upstream of the *FvCAMTA1* ATG start codon (**Figure 3A**). Bioinformatic analysis using NNPP database identified three potential promoter regions located at positions 148–198 bp, 925–975 bp, and 1,076-1,126 bp relative to the transcription start site. PlantCARE analysis revealed conserved core promoter elements including TATA-box (essential for transcription initiation) and CAAT-box (enhancer element), which conform to eukaryotic promoter structural characteristics. Functional motif analysis identified comprehensive regulatory elements: Hormone response elements, light response elements, abiotic stress elements, and MYB/MYC binding sites were identified (**Figure 3B**), suggesting complex regulatory mechanisms under both developmental and stress conditions.

**Figure 4:**
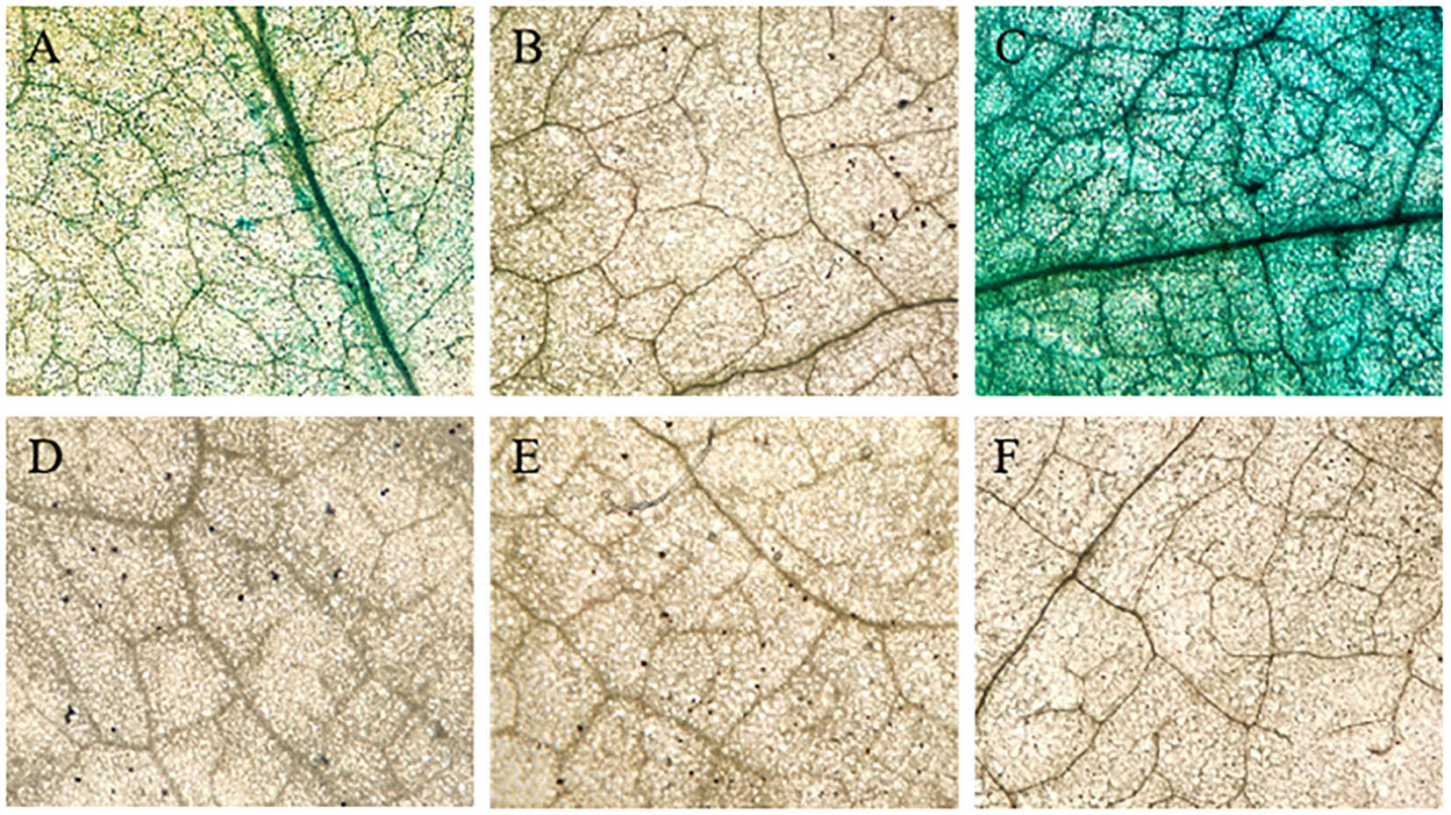
GUS staining of promoters of different lengths in tobacco leaf after salt stress treatment. **(A–C)** demonstrate the expression of FVCAMTA1::GUS constructs driven by promoter regions of 157 bp, 307 bp, and 1084 bp in tobacco leaves. **(D)** Represents the negative control injected with a blank vector. **(E)** depicts samples subjected to salt stress without blank vector injection. **(F)** Blank vector-injected controls under salt stress.

To investigate salt-responsive promoter regions, we performed Agrobacterium-mediated transient transformation of salt-stressed tobacco leaves. Three promoter fragments (−1,084 to +1: 1,084 bp; −307 to +1: 307 bp; −157 to +1: 157 bp) were cloned into pPZP211-GUS vector (**Figure 4**). GUS staining showed that the 157-bp fragment produced consistent blue staining in salt-treated leaves, indicating that this minimal region contains essential *cis*-elements (**Figure 4A**). The 1,084-bp fragment showed significantly deeper staining intensity compared with the 157-bp fragment under identical conditions (**Figure 4C**). In the 307-bp fragment and controls, there was no detectable GUS activity in either empty vector or buffer-treated controls (**Figures 4B, D–F**). This suggests that whereas the 157-bp core region maintains basic promoter functionality, upstream sequences (particularly between −1,084 and −157 bp) contain enhancer elements critical for full transcriptional activation under salt stress.

### Functional validation and of FvCAMTA1 in transgenic *Arabidopsis*

**Figure 5:**
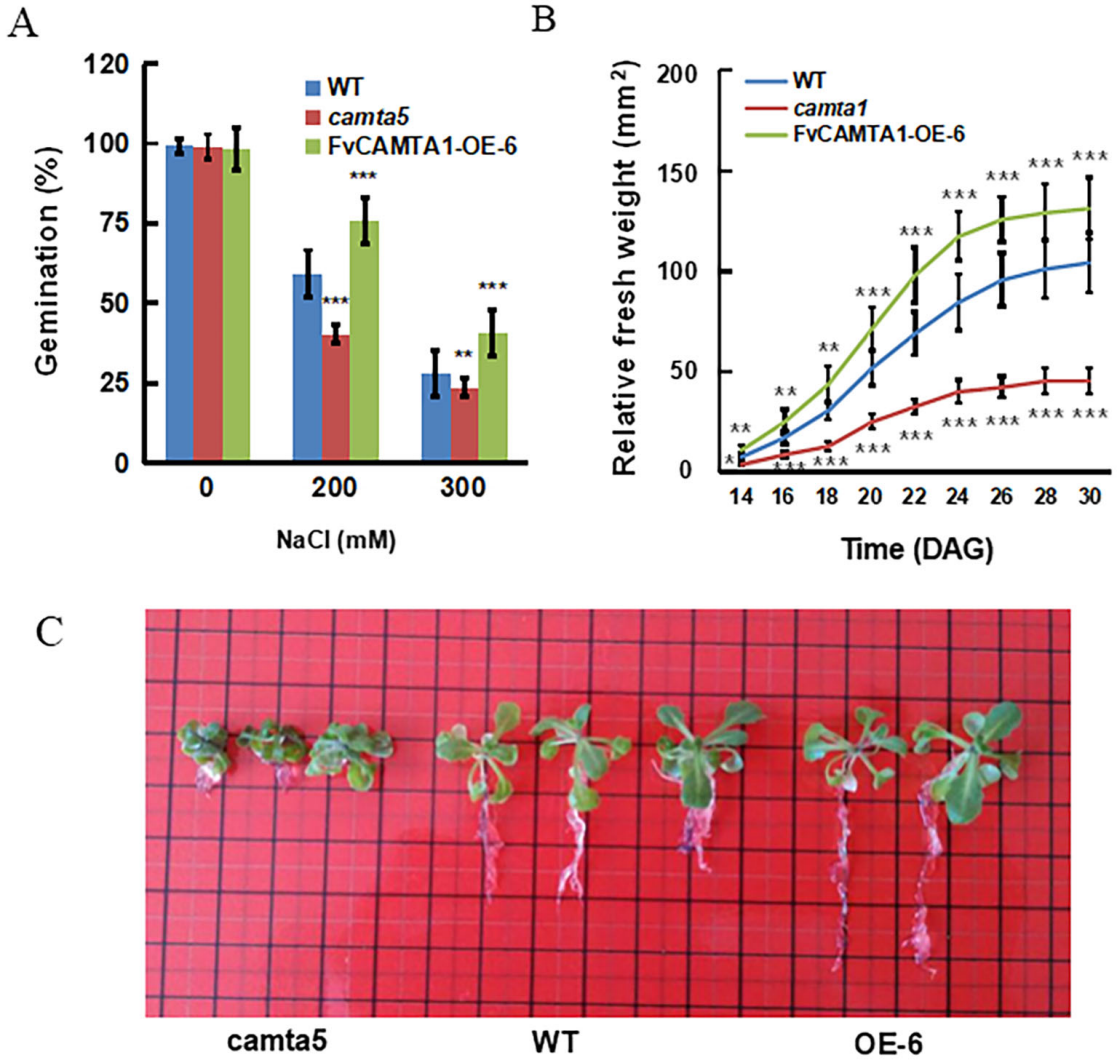
The salt-tolerant phenotypes of camta5 mutant and FVCAMTA1 overexpression plants **(A)** After 5 days of germination on 1/2 MS medium with different concentrations of NaCl, the germination number on 1/2 MS medium without NaCl was used as the control to calculate *the relative germination rate of different materials; **(B)** After germination on 1/2 MS medium without NaCl for 7 days, they were transferred to the nutrient soil and treated with a 200 mM NaCl aqueous solution for 14-30 days. The fresh weight of the aboveground part of the WT plants on the 30th day was used as the control to calculate the relative fresh weight at different times and for different materials. **(C)** The root phenotypes of different materials after 14 days of cultivation on 1/2 MS medium with 200 mM NaCl. Representation: mean ± standard deviation, *represents p< 0.05, **represents p<0.01, ***represents p<0.001, Student's t-test, n = 20.

To clarify the contribution of *FvCAMTA1* to salt tolerance, *FvCAMTA1* overexpression lines were generated through Agrobacterium-mediated transformation. Given 82% amino acid identity between *FvCAMTA1* and *Arabidopsis* AtCAMTA5, *camta5* mutants and wild type were selected as controls (**Figure 5**). Under 200 mM NaCl stress, germination rates at day 5 showed *camta5* mutants (42.3 ± 3.5%) significantly lower than the wild type (58.7 ± 4.1%, p<0.05), whereas the OE-6 line reached 72.6 ± 5.2% (**Figure 5A**). At 300 mM NaCl, *camta5* mutants (23.4 ± 2.8%) remained significantly lower than the wild type (29.7 ± 5.1%, p<0.05), with OE-6 at 45.3 ± 4.7%. Root phenotyping showed OE-6 primary root length (4.2 cm) significantly longer than the wild type (2.9 cm, p<0.01) under 200 mM stress, versus *camta5* mutants (1.1 cm) (**Figure 5C**). Long-term 200 mM NaCl treatment showed *camta5* mutants’ fresh weight lower than the wild type, whereas OE-6 exceeded wild-type levels (**Figure 5B**), confirming *FvCAMTA1*’s positive regulatory role in salt stress responses.

### *FvCAMTA1* majorly expressed in leaves and positively regulates resistance of salt stress

**Figure 6:**
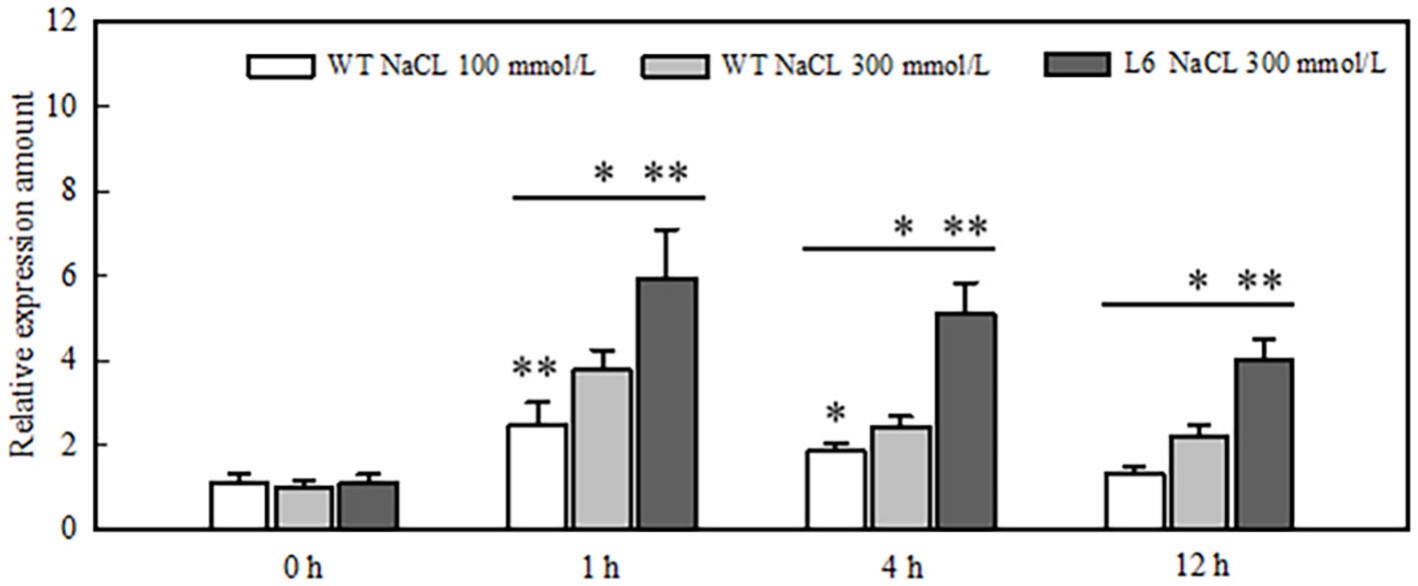
Expression of *FVCAMTA1* in WT and salt-tolerant cultivar ‘lila6' under 100 mmol/L and 300 mmol/L NaCl treatment. *represents a significant level of p<0.05, **represents a significant level of p<0.01.

To clarify the response of *FvCAMTA1* under salt stress, tissue expression specificity and salt stress treatment were performed. *FvCAMTA1* is expressed at the lowest level in the stem, followed by the root, which is 2.1 times that of the stem. It is expressed at the highest level in the leaves, being 3.2 times that of the stem (**Supplementary Figure 1**). Under the treatment of 100-mmol/L NaCl solution, the expression level of *FvCAMTA1* shows a trend of rapid increase followed by a decrease. At 1 h, the expression level of *FvCAMTA1* is the highest, 2.236 times that of 0 h (**Figure 6**). Then, it rapidly decreases and reaches the same level as 0 h at 12 h. Under the treatment of 300 mmol/L NaCl solution, the expression trend of *FvCAMTA1* is similar to that of 100 mmol/L NaCl treatment, but the expression level is significantly higher than that of 100 mmol/L NaCl treatment. At 1 h, its expression level is 3.8 times that of 0 h, and at 12 h, the expression level is still significantly higher than 0 h, being 4.1 times that of 0 h. In the salt-tolerant variety ‘Lu Xiaowu 6’, the expression level of *FvCAMTA1* is higher than that in ‘Qing Bi’. After 1 h of treatment with 300 mmol/L NaCl solution, the expression level of *FvCAMTA1* in ‘Lu Xiaowu 6’ is 6 times that of 0 h, and at 12 h, it is still 4.1 times that of 0 h (**Figure 6**).

### Yeast two-hybrid screening identified multiple interacting proteins *FvCAMTA1*

**Figure 7:**
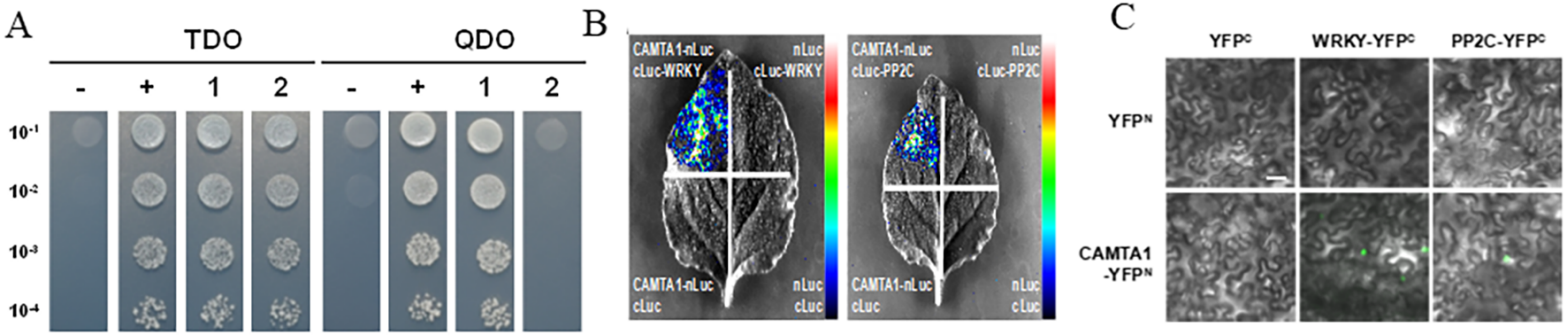
Y2H, LCA and BiFC analysis shown FVCAMTA1 interacts with FvWRKY7 and FvPP2C60. **(A)** Yeast two-hybrid (Y2H) assays confirmed interactions between FVCAMTA1 and FvWRKY7/FvPP2C60.TDO: SD/-Leu/-Trp/-His medium with 5 mM 3AT; QDO: SD/-Leu/-Trp/-His/-Ade medium; Numbers indicate dilution factors; "-": pGBKT7-FvCAMTA1+pGADT7 (negative control); "+": PGBKT7-53 +pGADT7-T (positive control); "1": pGBKT7-FvCAMTA1+pGADT7-FvWRKY7; "2": pGBKT7-FVCAMTA1+pGADT7-FvPP2C60. **(B)** Tobacco luciferase complementation assays (LCA) validated FVCAMTA1 interactions with FVWRKY7 and FvPP2C60. Upper-left quadrant: Agrobacterium mixture carrying pCAMBIA 1300-nLUC-FVCAMTA1 and pCAMBIA1300-cLUC-FvWRKY7/FvPP2C60; Upper-right and lower-left quadrants: Negative controls with one partner + empty vector (e.g., pCAMBIA 1300-nLUC-FvCAMTA1 + pCAMBIA1300- CLUC);Lower-right quadrant: Double empty vector control. **(C)** Bimolecular fluorescence complementation (BIFC) in tobacco epidermis demonstrated FVCAMTA1 interactions with FvWRKY7 and FvPP2C60.Row 1: PSPYNE-35S empty vector (negative control); Row 2: PSPYNE-35S-FVCAMTA1;Column 1: PSPYCE-35S empty vector (negative control);* Columns 2-4*: PSPYCE-35S-FvWRKY7, pSPYCE-35S-FvPP2C60;Scale bar = 50 μm.

To confirm how *FvCAMTA1* induced salt resistance, Y2H were performed (**Supplementary Figure 2**). A total of 46 interacting proteins were screened from cDNA library of salt-stressed *Fraxinus velutina* (**Supplementary Table 2**). To confirm Y2H screening results, Y2H analysis, BiFC analysis, and LCA were performed between *FvCAMTA1* and two genes: *FvPP2C60* and*FvWRKY7*. Y2H results showed that *FvCAMTA1* strongly interacted with *FvWRKY7* and weakly interacted with *FvPP2C60* (**Figure 7A**). LCA and BiFC analysis showed that *FvCAMTA1* interacted with *FvPP2C* and *FvWRKY7* (**Figures 7B, C**).

## Discussion

### Structural and promoter architecture of CAMTA1

CAMTA transcription factors are evolutionarily conserved across eukaryotes, with documented roles in *Populus trichocarpa* (Li et al., 2015), *Gossypium* (Zhang et al., 2016), and *Phaseolus vulgaris* (Büyük et al., 2019). Our study successfully cloned the full-length *FvCAMTA1* promoter containing typical eukaryotic promoter elements (TATA-box at −28 bp, CAAT-box at −98 bp) and multiple stress-responsive motifs. Functional validation confirmed that the 157-bp core promoter region maintains expression capability, whereas upstream elements enhance transcriptional activity. Galon et al. (2010a) demonstrated salt concentration-dependent GUS expression in *AtCAMTA1* promoter studies. Prasad et al. (2016) revealed CAMTA3/SR1’s regulatory roles in salt stress through RNA-seq. Our transient expression assays confirmed *FvCAMTA1*’s salt-responsive activation, with expression levels positively correlated with stress severity and cultivar tolerance, aligning with Büyük et al.’s (2019) findings in common beans.

The 1,231-bp *FvCAMTA1* promoter contains overlapping regulatory modules for hormonal crosstalk and abiotic stress. The 157-bp core region suffices for basal expression, but upstream elements (−1,084 to −157) amplify salt responsiveness. This modular organization resembles *AtCAMTA1*’s promoter, where auxin-responsive elements mediate thermotolerance (Galon et al., 2010a). The promoter sequence also contains plant hormone response element, light response element, and salt stress response element TC-rich repeats. Hormone response elements include salicylic acid (SA) response element TCA-element, abscisic acid (ABA) response element ABRE, jasmonic acid (JA) response element TGACG-motif and CGTCA-motif, ethylene response element ERE, and auxin response element TGA-element. SA, ABA, JA, and ethylene were reported to respond to salt stress (van Zelm et al., 2020; Zhang et al., 2022; Park et al., 2022; Liu et al., 2022; Gao et al., 2025). The abundance of ABRE and ARE motif implies integration with ABA-dependent and anaerobic pathways during salt adaptation.

### FvCAMTA1 positively regulates salt resistance

Transgenic Arabidopsis assays confirm *FvCAMTA1*’s positive regulatory role in salt tolerance. Enhanced germination and root growth in overexpression lines mirror *OsCAMTA4*’s function in rice (Ding et al., 2020) whereas *camta5* hypersensitivity highlights genetic specificity.

Expression levels of *FvCAMTA1* were induced by salt stress (**Figure 6**). In the salt-tolerant variety ‘Lu Xiaowu 6’, the expression of *FvCAMTA1* was significantly higher than that in wild-type ‘Qing Bi’ under salt stress. These results indicate that *FvCAMTA1* positively regulated salt resistance.

The promoter of *FvCAMTA1* in ‘Qing Bi’ and ‘Lu Xiaowu 6’ showed a motif difference (**Supplementary Figure 3**). MYB and TC-rich repeats were missing in ‘Qing Bi’, whereas the G-box was missing in ‘Lu Xiaowu 6’. These might result into a different response of FvCAMTA1 between two varieties.

### *FvCAMTA1* is expressed in leaves and might contribute to biotic stress

*FvCAMTA1* expression in leaves suggests roles in photosynthetic tissue protection, contrasting with root-predominant *AtCAMTA1*. However, shared drought response mechanisms exist: *FvCAMTA1* induction correlates with *AtCAMTA1*’s regulation of osmotic adjustment genes (Pandey et al., 2013). This functional overlap implies conserved stress response networks despite divergent expression gradients.

Y2H screening identified 46 interact proteins of FvCAMTA1. Among them, *FvWRKY7* and *FvPP2C60* were confirmed by Y2H, BiFC, and LCA. WRKY7 were members of the NRT1/PTR FAMILY 6.4, the WRKY7 transcription factor, ribosomal proteins, metallothionein, early light-induced protein (ELIP), and several proteins of unknown function. In *Myrothamnus flabellifolia*, the overexpression of MfWRKY7 exhibited a longer root length, better growth performance, higher contents of leaf water, and chlorophyll and osmolyte accumulation under salt stress compared with the wild type (Xu et al., 2025). Overexpression of *Arabidopsis thaliana AtWRKY7* enhanced resistance to Pseudomonas syringae and black spot disease (Kim et al., 2006); *OsWRKY7* was the potential candidate gene for resistance to panicle blast in rice (Sureshkumar et al., 2019). The interaction between *FvCAMTA1* and *FvWRKY7* suggests that they may cooperatively regulate plant salt responses. Furthermore, *FvCAMTA1* was found to interact with *FvPP2C60*. In *Arabidopsis thaliana*, protein phosphatase 2C (PP2C) participates in the ABA signaling pathway and positively regulates salt stress (Yang et al., 2024).

In conclusion, we report the functional characterization of *FvCAMTA1* in woody plants, demonstrating its dual role as a calcium sensor and transcriptional activator in salt stress networks. Comprehensive promoter analysis and transgenic validation collectively establish *FvCAMTA1* as a positive regulator of salt tolerance in *Arabidopsis thaliana*. These findings provide molecular insights into CAMTA-mediated stress adaptation and offer biotechnological potential for forest tree improvement The function of FvCAMTA1 in salt stress response will be further verified in *Fraxinus velutina* in future studies.

## Data Availability

The original contributions presented in the study are included in the article/**Supplementary Material**. Further inquiries can be directed to the corresponding author.

## Supplementary material

Supplementary Table 1 Primers used in experiment.

Supplementary Table 2 Genes screened by Y2H
