# Supplementary figures and images for: Functional characterization of FvCAMTA1in salt stress response of Fraxinus velutina
Source: Front Plant Sci. 2025 Oct 29;16:1669043. doi: 10.3389/fpls.2025.1669043 (PMC12608651; doi:10.3389/fpls.2025.1669043)

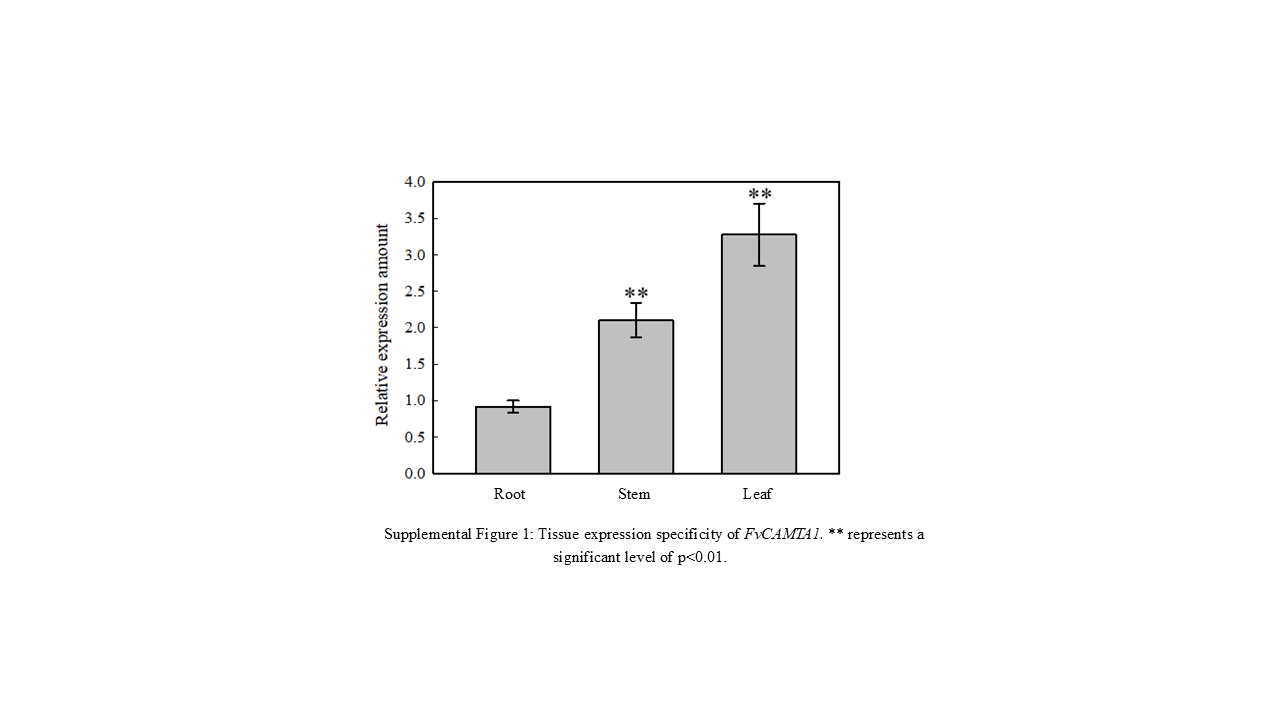

Supplement: Supplementary file 3 [file Image1.tif]

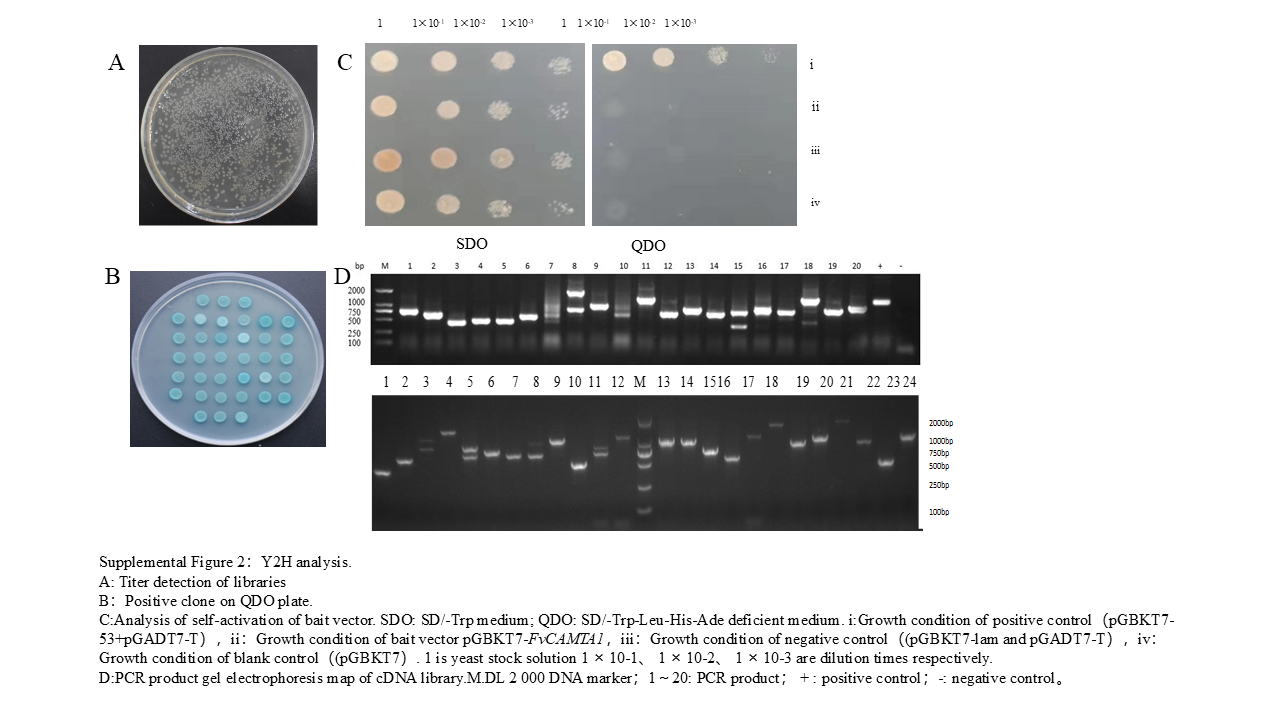

Supplement: Supplementary file 4 [file Image2.tif]

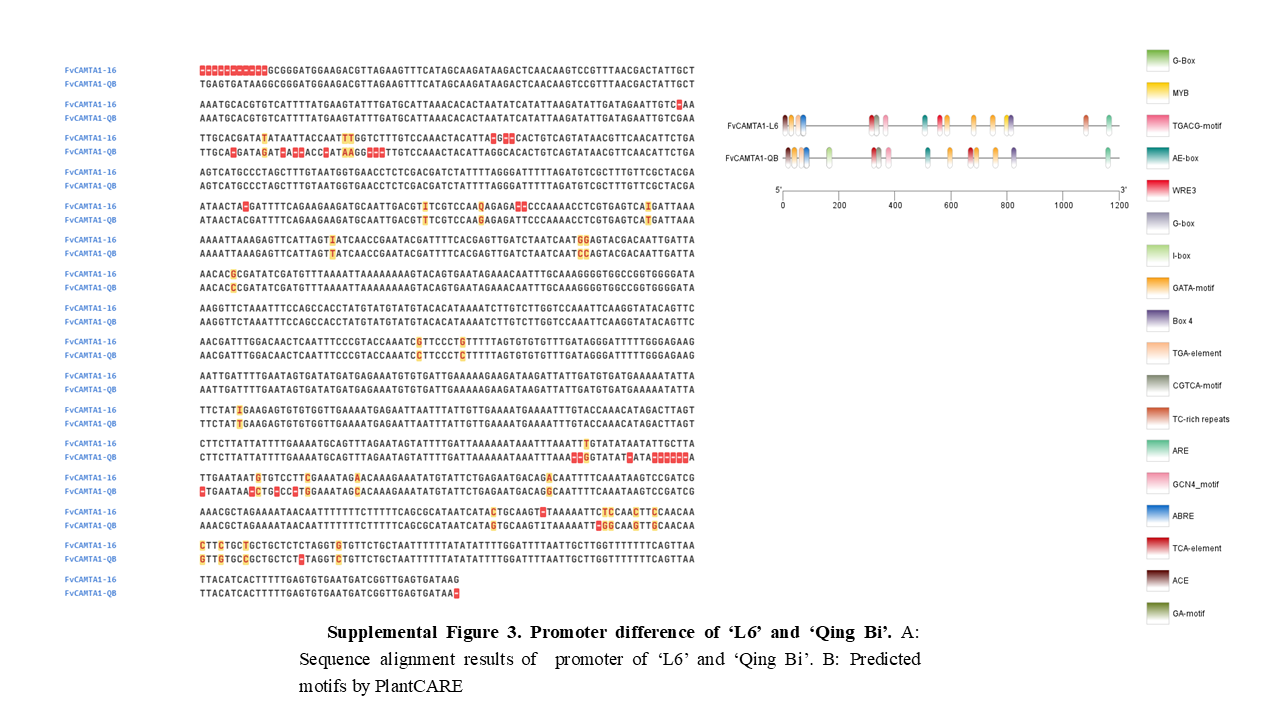

Supplement: Supplementary file 5 [file Image3.tif]
